# The Morphological Image of Fat Body and Tergal Gland Cells in Uninseminated *Apis mellifera* Queen Bees

**DOI:** 10.3390/insects15040244

**Published:** 2024-04-03

**Authors:** Milena Jaremek, Krzysztof Olszewski, Jacek Chobotow, Aneta Strachecka

**Affiliations:** 1 Department of Invertebrate Ecophysiology and Experimental Biology, Faculty of Environmental Biology, University of Life Sciences in Lublin, Doświadczalna 50a, 20-280 Lublin, Poland; aneta.strachecka@up.lublin.pl; 2 Subdepartment of Apidology, Institute of Biological Basis of Animal Production, Faculty of Animal Sciences and Bioeconomy, University of Life Sciences in Lublin, Akademicka 13, 20-950 Lublin, Poland; krzysztof.olszewski@up.lublin.pl; 3 Faculty of Biology and Biotechnology, Maria Curie-Sklodowska University, Akademicka 19, 20-400 Lublin, Poland; jacek.chobotow@poczta.umcs.lublin.pl

**Keywords:** honeybees, reproductive caste, tissues, microscopy, age, oenocytes, fat body

## Abstract

**Simple Summary:**

The importance of research on honeybee queen immunity is underscored by the fact that it is the only reproductive caste in the bee colony, whose genetic and biological quality largely translates into the condition and productivity/efficiency of the colony. Queen bee fat body and tergal glands play crucial roles in bee immunity. The role of the fat body is not limited to storing energy reserves in the form of fat, protein, or glycogen. This tissue has detoxifying functions and participates in the maintenance of homeostasis. Additionally, the secretion of tergal glands allows workers to recognize the queen and is an attractant for drones during mating flights. The characteristics of tergal glands in queens are important for understanding the biology of reproduction. We determined changes in the morphological image of fat body and tergal gland cells in uninseminated *Apis mellifera* queen bees. We showed that the segmental character of the fat body depends on its location, not only in 1-day-old queens, but also in those at 8 and 20 days of age. The morphological image of tergal gland cells was not different in the queens aged 1 and 8 days, but dark-colored lumps appeared in the cells of those aged 20 days.

**Abstract:**

The morphological changes in fat body cells, tergal gland cells, and the surface areas of the cell nuclei were determined in queen bees of the subspecies *Apis mellifera carnica*. This study focused on 1-, 8-, and 20-day-old uninseminated females kept in colonies, analyzing cells from three locations in the abdomen: the sternite, and tergites III and V. The oenocytes in the sternites were large, oval/circular with a centrally located nucleus, while in tergites III and V, they were small and triangular in the 1-day-old queens. During the first week of life, these cells in tergites III and V change their shape to oval and increase their sizes. The initially light yellow and then dark yellow granularities in the oenocytes of the fat body appear along with the advancing age of the queens. The trophocytes (sternites, tergites III and V) in the 1-day-old queens were completely filled with droplets of different sizes. In the 8- and 20-day-old queens, the number and size of the droplets decreased in the trophocytes of tergites III and V. The tergal gland cells had a centrally located cell nucleus in the 1-, 8- and 20-day-old queens. The dark granularities in these cells were visible only in the 20-day-old queens. Different morphological images of the fat body at the sternite, and tergites III and V, and the difference in the size of the oenocyte cell nuclei may indicate various functions of the fat body depending on its location. Characterization of the changes in the morphology of the fat body, taking into account its segmental character, and the tergal glands requires further research in older queens, e.g., one-year-old, brooding queens.

## 1. Introduction

Honeybees are insects that live in two environments: in the colony, the conditions of which they strictly control, and in the changing conditions of the natural environment. The ability to regulate the internal conditions of the colony translates into a significant independence from the changing conditions of the external environment [1]. The conditions in the colony directly affect the queen bee. The queen bee is also indirectly affected by external environmental factors, including those of anthropogenic origin, e.g., chemicalization of agriculture, pesticides, lack of a nutritious/complete diet, monodiet, pathogens. These factors can contribute to a decrease in bee immunity [2,3,4,5]. A high density of individuals, and high humidity and temperature, make both adult insects and their developmental forms vulnerable to pathogen activities. Under such conditions, only the high efficiency of immunity mechanisms ensures the survival of bees [6,7,8,9]. The penetration of toxic compounds or pathogens into the hemolymph disrupts metabolism and affects the immune protein synthesis pathways [5].

So far, research on bee immunity has focused mainly on the workers [10,11,12], while little attention has been paid to queen immunity. The importance of research on queen immunity is emphasized by the fact that the queen is the only reproductive female in the bee colony, whose genetic and biological quality highly influences the condition and productivity of the colony.

Nowadays, it is known that the fat body and tergal glands are tissues which play a crucial role in bee immunity [13,14,15]. The role of the fat body is not limited to storing energy reserves in the form of fat, protein, and glycogen. This tissue has detoxifying functions and participates in the maintenance of homeostasis. In this tissue, oenocytes and trophocytes are the predominant cell types. Trophocytes are cells with lipid drops distributed over the entire cell surface in 1-day-old queens and on the periphery in 1-day-old workers. Intercellular spaces are visible in the workers. In contrast, the cells are tightly attached to each other in the queens. The trophocytes in the fat bodies from the third tergites are especially large in the queens in comparison to the other locations [13,15]. Oenocytes are circular, oval, elliptical or triangular cells that are distributed between the trophocytes in the different segments of the fat body. They are cells with a centrally located cell nucleus, around which other organelles are located, i.e., mitochondria, endoplasmic reticulum, and vacuoles filled with lipids and glycogen. There are no oenocytes in the fat body located at the third tergite in the workers, but there are a lot of them in the queens [13,15]. Oenocytes are responsible for the synthesis of hormones, as well as sex pheromones. Therefore, their presence in queens may indicate an adaptation for reproductive function, as compared to infertile/sterile workers. The number, size, and color of oenocytes depend on their location in the fat body, the developmental stage, age, and caste of the bee. In young bees, the oenocytes are small, sparse, and yellowish, while in flying bees, they are large, numerous, and amber-colored or sometimes brown. Most of the proteins in a bee’s hemolymph are synthesized in the fat body [13]. A large proportion of these proteins are antimicrobial peptides, which are an important component of humoral immunity of bees. Vitellogenin (Vg) is synthesized in the trophocytes. Vg has antioxidant properties and regulates oogenesis. In the bee body, Vg is captured by specific groups of cells and is involved in various physiological processes, e.g., stress reduction, lifespan regulation, reproduction; metabolic processes, e.g., regulation of juvenile hormone concentration; and behavioral processes, e.g., feeding. The largest amount of vitellogenin is produced by the fat body of nurse bees, but even then it is only 1/20th of the queen bees’ vitellogenin levels [9,13,16,17,18,19,20]. The number of eggs laid by the queen depends on the concentration of Vg. Therefore, the more efficient the “working cells”/trophocytes, the more Vg there will be, and the more eggs the queen will lay. The situation in the colony also adds to this. The quality of the fat body depends on the diet in the first days after emergence. If there is food supply in the colony (mainly pollen), then the nurse bees will produce royal jelly which the queens will be fed, and then such queens will be healthy and strong, and their fat body will function properly and produce of protein i.a. Vg [9,13,21,22].

Fat body cells of *A. mellifera* females are located next to the tergal gland cells and provide them with metabolically necessary compounds thus influencing their function. Tergal glands in queen bees are located on the underside of the third, fourth, and fifth tergites in the dorsal part of the abdomen. The queens have a lot of the cells (there are 25–32 glandular cells on the 200 µm^2^ tissue surface) that are closely adhered to one another, with a centrally located nucleus. From each cell departs the outlet ducts, from which pheromones are emitted with pulsating movements. No differences are observed in the morphological images between the glands from various tergites (III, IV, and V), but these glandular cells differ in their nucleus diameters—the largest nuclei are found in the third tergite and the smallest ones in the fifth [14]. In order for the queen to be valuable and perform her functions, the tergal gland cells should be compact and in adequate numbers. The reproductive status of the queen depends on these parameters. Therefore, the determination of factors that affect the quality of these cells, especially in young queens, has not only a cognitive but also an economic dimension in apiary management. The secretion of the tergal glands allows workers to recognize the queen and is an attractant for drones during mating flights [23,24,25]. Therefore, the characteristics of the tergal glands in queens preparing for the mating flight are important for understanding the biology of reproduction of these important pollinators.

The aim of the research was to determine changes in the morphological image of fat body and tergal gland cells, and the metabolic activities of the fat body oenocytes and tergal gland cells in *A. mellifera* queen bees at emergence (1-day-old) and sexual maturity, when they are ready for the mating flight and insemination (8-day-old), as well as those detained maximally in colonies without insemination (e.g., in the absence of favorable weather conditions for the mating flight; 20-day-old).

## 2. Materials and Methods

This study was performed at the apiary of the University of Life Sciences in Lublin, Poland (51.224039° N–22.634649° E).

### 2.1. Experimental Design

The biological material was queen honeybees of the subspecies *Apis mellifera carnica*; 1-, 8- and 20-day-old uninseminated females kept in colonies. The queens were reared from larvae obtained from the same colony of Carnica bees, from an artificially inseminated reproductive queen. In order to obtain larvae of a similar age, four days before the planned rearing, the reproductive queen was confined in a queen-excluder comb-cage, containing one empty comb, for 24 h to lay eggs. One day after being placed in the queen-excluder comb-cage, the queen was released, while the brood comb remained. On the fourth day from that day, the queen was confined on a comb placed in the queen-excluder comb-cage, the comb was taken out and the larvae were transferred from it onto royal jelly diluted with water in queen cell cups [26]. The royal jelly used in the rearing of all the queens that came from the same batch from one rearing colony. The royal jelly from all the queen cells was collected into one vessel, after which water was added to it according to Büchler et al.’s [26] method [a mixture of half royal jelly and half warm water]. Following this, the royal jelly and water were mixed to a uniform consistency and used for queen larvae rearing. The frames with transferred larvae were placed in very strong queenless rearing colonies in Dadant hives. On the seventh day, the queen cells were secluded in isolators and put in an incubator (temperature 35 °C, relative humidity 60%) until they emerged. On the twelfth day (counting from the transfer of larvae) the isolators with emerged queens were removed from the incubator. A total of 10 of the 1-day-old queens were taken for analysis, while the rest were placed in colonies and kept there until the 8th (group: 8-day-old, 10 queens) and 20th (group: 20-day-old, 10 queens) day of their life. In order to prevent the queens from an insemination/mating flight, the hive entrance was fenced with a queen-excluder. This allowed us to evaluate the effect of age, while eliminating the effect of change in the reproductive status.

### 2.2. Morphological Analyses

#### 2.2.1. The Morphological Image of the Fat Body and Tergal Gland Cells

The fat bodies from the sternites (between the third and the fifth sternites), and the third and the fifth tergites in each of the 30 queen bees, 10 from each age group (1-, 8- and 20-day-old) were dissected under a stereoscopic microscope (Olympus SZ60, Olympus Corporation, Tokyo, Japan). The dorsal segments were selected due to their immediate proximity with tergal glands. The tergal gland tissues were also dissected. Microscopic preparations were made from the dissected tissues. The tissue fragments were placed on glass slides in 0.6% NaCl and covered with a cover-glass. Microscopic preparations were observed and the fat body and tergal gland cells were photographed with an Olympus DP 72 camera (Microscope Olympus BX61, Olympus Corporation, Tokyo, Japan; magnification × 40) with a DIC attachment.

#### 2.2.2. The Surface Area of the Cell Nuclei of the Fat Body Oenocytes and Tergal Gland Cells

The measurements of the surface areas of nuclei in the living cells, the oenocytes and the tergal gland cells, were taken at 40× magnification. Microscopic preparations were observed and the fat body and tergal gland cells were photographed with the Olympus DP72 camera (Microscope Olympus BX61; 40× magnification) with the DIC attachment. Visualization of the living (undistorted) tissues/cells was made according to the Strachecka et al. [15,27] methods. For each location of the fat body (sternites, tergite III, and tergite V) of the queen age groups (1-, 8- and 20-day-old), 10 nuclei of the oenocytes and tergal gland cells were measured. By operating the micrometer screw before measurement, the microscopic image was set so that the nucleus showed the largest possible area. A total of 100 measurements (10 queens × 10 measurements) were performed for each fat body location and tergal gland in each age group.

### 2.3. Statistical Analysis

The statistical analysis of the results was carried out using Statistica software formulas, version 13.3 (2017) for Windows, StatSoft Inc., Tulsa, OK, USA. The distribution of the data were analyzed with the use of the Kolmogorov–Smirnoff test. The effect of the queens’ age (1-, 8- and 20-day-old) on the surface areas of the fat body oenocytes for tergites III (n = 300 measurements) and tergites V (n = 300 measurements) was assessed with the Kruskal–Wallis test (data with non-normal distribution), and for sternites (n = 300 measurements), ANOVA was used (data with normal distribution). The effect of fat body locations (sternites—n = 100 measurements, tergites III—n = 100 measurements, and tergites V—n = 100 measurements) on the surface areas of the fat body oenocyte nuclei for each age group of queens was assessed with the Kruskal–Wallis test (data with non-normal distribution).

The surface areas of the fat body oenocyte nuclei between the queens’ age groups within each location (sternites—n = 100 measurements, tergites III—n = 100 measurements, and tergites V—n = 100 measurements) were compared for tergites III and tergites V with the Mann–Whitney U test (data with non-normal distribution), and for sternites with Tukey’s HSD test (data with normal distribution).

The surface areas of the fat body oenocyte nuclei between locations (sternites—n = 100 measurements, tergites III—n = 100 measurements, and tergites V—n = 100 measurements) within each queen age group were compared with the Mann–Whitney U test (data with non-normal distribution).

The effect of the queens’ age on the surface area of the tergal gland cell nuclei (1-day-old—n = 100 measurements, 8-day-old—n = 100 measurements and 20-day-old—n = 100 measurements) was evaluated with the Kruskal–Wallis test (data with non-normal distribution), and between the queen age groups, it was compared with the Mann–Whitney U test.

## 3. Results

### 3.1. The Morphological Image of the Fat Body Cells and the Surface Area of the Cell Nuclei of Fat Body Oenocytes

#### 3.1.1. The Morphological Image of the Fat Body Cells

In the queens, the sizes, shapes, and arrangement of fat body cells differ between segments, indicating the segmental character of the fat body (Table 1A–C).

The changes in the oenocytes:

- in the sternites: the cells are large, oval/circular with a discernible centrally located nucleus;
- in tergites III and V:
  -> in the 1-day-old queens, the cells are small and triangular, then change their shape to round/oval and increase in size during the first week of life;
  -> in the 8- and 20-day-old queens the cell nuclei are visible;
- in the fat body: in the sternite, and tergites III and V, the process of vacuolization is visible in the 8-day-old queens;
- as the queen matures, yellow granules appear, which are especially visible in the 20-day-old queens.

The changes between the 8- and 20-day-old queens were not so clear and mainly concerned the oenocytes, in which the abovementioned granularities changed their color from light yellow to dark yellow.

The changes in the trophocytes:

- in the 1-day-old queens the cells are completely filled with droplets of different sizes in each of the locations of the fat body,
- in tergites III and V, the number and size of the droplets decreases, and their position changes from all-over to the edge; in the sternites, these changes occur more slowly.

#### 3.1.2. The Surface Area of the Cell Nuclei of the Fat Body Oenocytes

The queens’ age (1-, 8- and 20-day-old) had a statistically significant effect on the size of the oenocyte nuclei in all the fat body locations (sternites—F_2, 279_ = 88.16, *p* = 0.00; tergites III—H = 147.75, df = 2, *p* ≤ 0.01; tergites V—H = 151.84, df = 2, *p* ≤ 0.01). In each age group of the queens, the location of the fat body (sternites, tergites III and tergites V) had a statistically significant impact on the size of the oenocyte nuclei (1 day—H = 229.19, df = 2, *p* ≤ 0.01; tergite III—H = 12.35, df = 2, *p* ≤ 0.01; tergite V—H = 59.75, df = 2, *p* ≤ 0.01).

**Figure 1:**
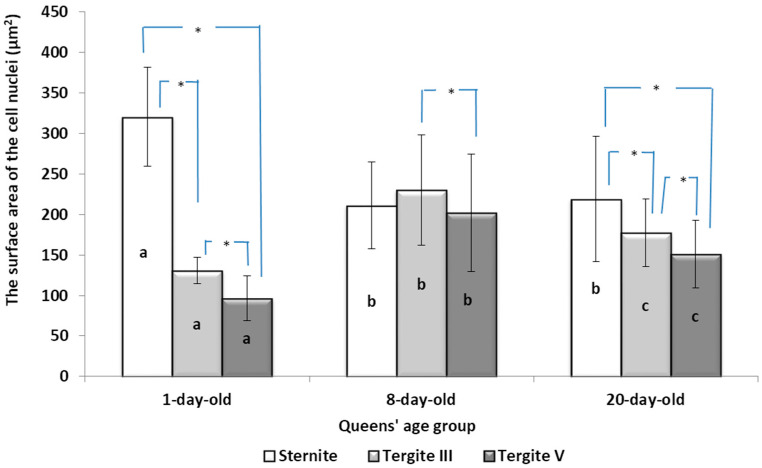
The surface area of the cell nuclei (µm^2^) of the fat body oenocytes depending on their location (µm; mean ± SD) in the three age groups of queens; (n = 3 age groups × 10 queens × 10 measurements); a, b, c—differences between the queen age groups (1-, 8- and 20-day-old) within the fat body locations are significant at *p* ≤ 0.01; *—differences between the fat body locations (sternites, tergites III and tergites V) within the queen age groups are significant at *p* ≤ 0.01; vertical bars indicate standard deviation.

Differences in the size of the oenocyte cell nuclei, related to the metabolic activities, were significant in all the queen age groups (Figure 1). This is evidenced by the reduction in the surface areas of the fat body oenocyte nuclei located at the sternites and the increase at tergites III and V in 8- and 20-day-old queens compared to 1-day-old queens. There were no statistically significant differences at the sternites between 8- and 20-day-old queens. Moreover, statistically significant differences were found between the fat body locations in 1- and 20-day-old queens. However, in the 8-day-old queens, these differences were statistically significant only between tergites III and V (Figure 1).

### 3.2. The Morphological Images and Surface Area of Cell Nuclei of the Tergal Gland Cells

#### 3.2.1. The Morphological Images of the Tergal Gland Cells

In all the age groups of queens (1-, 8- and 20-day-old), the round tergal gland cells with a centrally located cell nucleus were visible, while the dark granularities in the cells were visible only in the 20-day-old queens (Table 2).

#### 3.2.2. The Surface Area of the Cell Nuclei of the Tergal Gland Cells

The queens’ age (1-, 8- and 20-day-old) statistically significantly affected the surface areas of the tergal gland cell nuclei (H = 48.07, df = 2, *p* ≤ 0.01).

**Figure 2:**
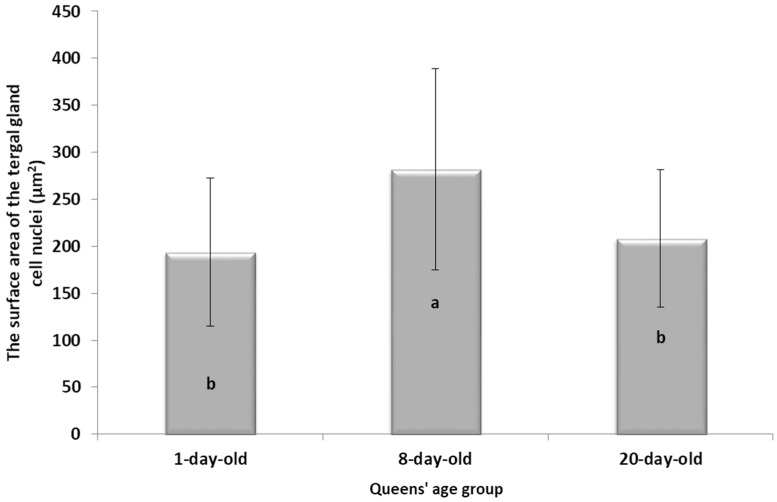
The surface area of the tergal gland cell nuclei (µm^2^) (mean ± SD) in the three age groups of queens; a, b—statistically significant difference at *p* ≤ 0.01 between the age groups of queens; vertical bars indicate standard deviation.

In the 8-day-old queens, the activities of the tergal gland cells were statistically significantly higher than in the 1- and 20-day-old insects (Figure 2). There was no statistically significant difference between the 1- and 20-day-old queens (Figure 2).

## 4. Discussion

### 4.1. The Morphological Images of Fat Body Cells and the Surface Area of the Oenocyte Cell Nuclei

Strachecka et al. [15] showed that the subcuticular fat body has a segmental character in 1-day-old *Apis mellifera* females and depends on different reproductive potentials. In this study, the authors focused on determining the morphological changes in the fat bodies and tergal glands in young and uninseminated queens, in the early stages of their lives (1–20 days), during which crucial processes to the queens’ continued functioning take place.

The authors of this study confirmed the segmental character of the fat body not only in 1-day-old queens but also observed the same trend in the insects aged 8 and 20 days. This segmental character means that the morphology (Table 1A–C) and activity (Figure 1) of the fat body depends on its sector. For example, oenocyte activity was particularly pronounced in the 1- and 20-day-old queens (Figure 1). The largest reserves of lipid, proteins, and carbohydrates stored in the fat body, in the form of a large number of lipid droplets distributed over the entire surface of the trophocytes, were found in the queens at the age of 1 day (Table 1A–C—1-day-old). This corresponds with the findings of the studies by de Oliveira and da Cruz-Landim [28] conducted on the workers of the *Melipona quadrifasciata anthidioides* species. In the trophocytes of the queen bees aged 8 days, the amount of fat was much lower, and lipid droplets were located only along the edges of cells (Table 1A–C—8-day-old). These changes suggest a consumption of energy reserves [22], presumably during the process of puberty and preparation for the mating flight of young queen bees. Yellow granularities were observed in the oenocytes of the 8-day-old queens (Table 1A–C—8 day-old-queens) and their color changed to dark yellow when the queens turned 20 days of age (Table 1A–C—20-day-old). We suggest that the granularities in question were the effect of the accumulation of metabolites. Münch et al. [29] suggested that this may be connected with the onset of aging processes in the organism. However, the queens aged 20 days cannot be treated as old, as queens can live for several years [30]. It can be assumed that the yellow granularities in the oenocytes are the result of a significant increase in the metabolic activities of the fat body oenocytes in the initial period of a queen’s life. The changes in the morphological image of the fat body and surface area of oenocytes were the most dynamic between the 1-, 8- and 20-day-old queens (Table 1A–C and Figure 1). The differences between the queens aged 8 and 20 days only at the sternites were not significant (Table 1). Moreover, the surface areas of the nuclei of the fat body’s oenocytes located at the tergites of the 8- and 20-day old queens were bigger than in those at the age of 1 day (Figure 1) and this may indicate the higher metabolic activity of the tissue [22]. Oenocytes are the equivalent of hepatocytes in mammals; they are involved in the synthesis of proteins, fats, hydrocarbons, and pheromones, which are transported to the surface of the cuticle, and are also used during the detoxification of the organism [15,20]. The metabolic activities of the cell nuclei expressed by their surface area depend on the caste and age of the bees, cell location and type of tissue [9,15,31,32], which is also confirmed by our research.

### 4.2. The Morphological Images of Tergal Gland Cells and the Surface Area of the Tergal Gland Cell Nuclei

We wondered if and how the morphology of the tergal glands would change in the three age groups of queen bees. We analyzed the literature and did not find any studies devoted to the analysis of the tergal glands in queen bees on such a detailed level. The results of Strachecka et al. [14] described the morphology of tergal glands in rebel workers, which are primed to reproduce rather than participate in the rearing of the next generation of sister–queen offspring. We showed what the tergal gland cells look like in 8-day-old queens. Therefore, we focused on presenting the morphological changes that occur in this tissue in queens between days 1 and 20, during the period when key processes for the queens’ continued functioning take place.

While the morphological image of the tergal gland cells was not different between the queens aged 1 and 8 days, in those at the age of 20 days, the dark-colored lumps in the cells had appeared (Table 2—20-day-old). According to the authors of this study, these are, in all likelihood, melanotic lumps developed as a result of the accumulation of large amounts of eumelanines, i.e., products of melanization, which protect the chitinous covering from pathogen penetration. This suggests better protection of the organism against the impact of external environmental factors [11,14,25,29,33,34]. The authors did not encounter a description of these lumps in the tergal gland tissue of queen bees in the literature, which suggests that we were the first to notice them. In worker bees, such dark-colored lumps are present in the trophocytes of the fat body and in the Nasonov gland. While the lumps in the trophocytes contain iron, calcium, magnesium, and phosphate ions [14,35], the lumps in the Nasonov gland are a cluster of melanin, proteins, or ions of metals that are accumulated in cells as a result of the aging processes in the organism, defense reactions and environmental factors [11,33,34,36,37,38]. The tendencies in the changes in surface area of the tergal gland cells of the queen bees corresponded with the changes in surface area of the fat body located at the tergites. For example, in the queens aged 8 days, both the surface area of tergal gland cells and that of the fat body sectors located at the tergites was higher than on the first day of the queens’ life (Figure 1 and Figure 2). The parallel between the surface area of the tissues and their metabolic activities in question can suggest a functional link between the tergal glands and the fat body. Moreover, the highest metabolic activities of tergal gland cells in the queens aged 8 days may be connected with the mating flights they do at this age; during the flights, the secretion of those glands is a factor that attracts drones [39,40,41,42,43]. Although the queen had limited possibilities of leaving the hive during our experiment, biochemical reactions were occurring in the queens’ bodies that enabled the queens to prepare for this activity.

The changes occurring in the young queens between the 1st and the 20th day of their lives were fast. It is worth emphasizing that these queens were uninseminated and did not lay eggs. So, the question arises whether similar changes will be observed in inseminated queens? What will the fat body and tergal glands look like in older queens, e.g., one-year-olds, two-year-olds? These questions set out the topics for future research.

## 5. Conclusions

The segmental character of the fat body depends on its location, not only in 1-day-old queens, but also in those at 8 and 20 days of age. The morphological image of tergal gland cells was not different in the queens aged 1 and 8 days, but dark-colored lumps appeared in the cells of those aged 20 days. In the trophocytes of the queen bees aged 8 days, the amount of fat was much lower, and lipid droplets were located only along the edges of the cells. These changes suggest a consumption of energy reserves, presumably during the process of puberty and preparation for the mating flight of young queen bees. The surface areas of the nuclei of the fat body’s oenocytes located at the tergites of the 8- and 20-day old queens were bigger than in those at the age of 1 day, which additionally confirms, and this may indicate the higher metabolic activity of the tissue. The tendencies in the changes in the surface area of metabolic activities in the tergal gland cells of the queen bees corresponded with the changes in the surface area of metabolic activities taking place in the fat body located at the tergites.

## Figures and Tables

**Table 1:** The morphological images of fat body cells depending on their location: at sternites (A), at tergite III (B), at tergite V (C) in 1-, 8- and 20-day-old queens (magnification × 40).

1-Day-Old	8-Day-Old	20-Day-Old
(A) Fat body location at the sternites		
** 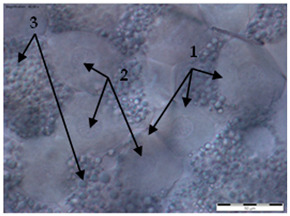 **	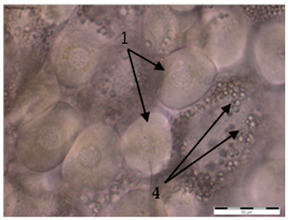	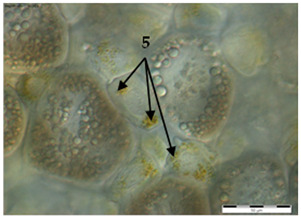
(B) Fat body location at tergite III		
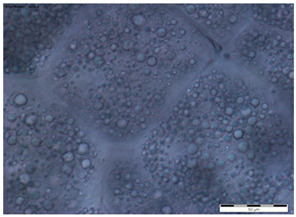	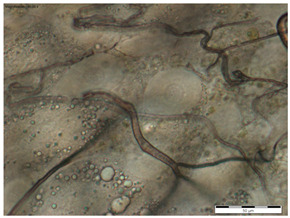	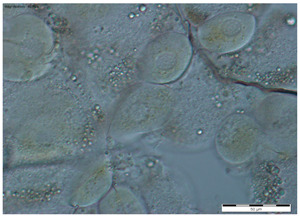
(C) Fat body location at tergite V		
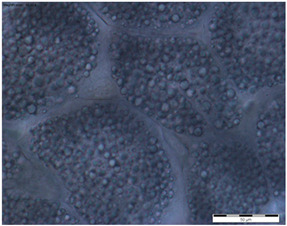	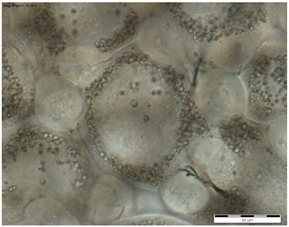	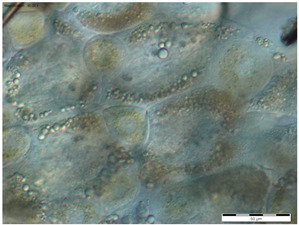

Explanations: 1—oenocyte; 2—oenocyte cell nuclei; 3—trophocyte; 4—droplets; 5—yellow granules; the scale bar in each photo is 50 µm.

**Table 2:** The morphological images of the tergal gland cells in the 1-, 8- and 20-day-old queens (magnification × 40).

1-Day-Old	8-Day-Old	20-Day-Old
The tergal gland cells		
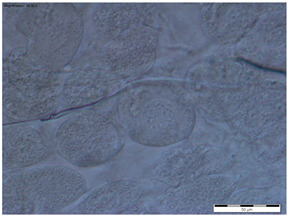	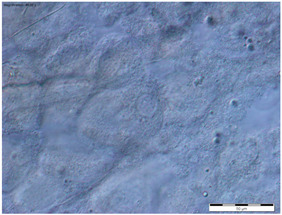	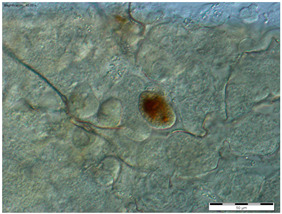

Explanations: the scale bar in each photo is 50 µm.

## Data Availability

The data sets generated during and/or analyzed during the current study are available from the corresponding author on reasonable request.
